# A retrospective analysis of the InterTan nail and proximal femoral nail anti-rotation-Asia in the treatment of unstable intertrochanteric femur fractures in the elderly

**DOI:** 10.1186/s13018-016-0344-7

**Published:** 2016-01-15

**Authors:** Weiguang Yu, Xinchao Zhang, Xingfei Zhu, Jun Hu, Yunjiang Liu

**Affiliations:** Department of Orthopedics, The First Affiliated Hospital of Sun Yat-sen University, Huangpu East Road No. 183, Huangpu District, Guangzhou City, Guangdong Province 510700 China; Department of Orthopaedics, Jinshan Hospital, Fudan University, Longhang Road No.1508, Jinshan District, Shanghai City, 201508 China

## Abstract

**Background:**

The purpose of this study was to compare the clinical outcomes of elderly patients undergoing surgery for treatment of unstable trochanteric fractures receiving either proximal femoral nails anti-rotation-Asia (PFNA-IIs) or InterTan nails (ITs).

**Methods:**

Between January 1, 2012, and June 31, 2015, 168 elderly patients with unstable intertrochanteric femur fractures enrolled in this study. The only intervention was ITs or PFNA-IIs of the unstable trochanteric femur fractures. Follow-up was at 1, 3, 6, and 12 months postoperatively and yearly thereafter. Intraoperative variables and postoperative complications were compared between the two groups.

**Results:**

Eight patients died, six were too infirmed for follow-up, and seven were lost during follow-up, leaving 147 patients meeting the criteria were evaluated at a mean follow-up of 20 months (range 16–26 months). Significant differences were observed between the two groups regarding local complications (IT, *n* = 10 vs. PFNA-II, *n* = 20), varus collapse of the head/neck or femoral shaft fractures at the tip of the nail (IT, *n* = 1 vs. PFNA-II, *n* = 8), femoral neck shortening (IT, 4.4 ± 1.1 mm vs. PFNA-II, 7.4 ± 2.4 mm), fracture healing time (IT, 14.7 ± 2.1 weeks vs. PFNA-II, 15.7 ± 2.4 weeks), femoral shaft fractures (IT, *n* = 0 vs. PFNA-II, *n* = 4), rotational loss of reduction (IT, *n* = 0 vs. PFNA-II, *n* = 9), lateral cortex fractures of the proximal femur or lateral greater trochanter fractures (IT, *n* = 8 vs. PFNA-II, *n* = 1), operative time (IT, 71.9 ± 6.8 min vs. PFNA-II, 52.3 ± 4.0 min), intraoperative blood loss (IT, 190.6 ± 6.0 mL vs. PFNA-II, 180.9 ± 10.8 mL), fluoroscopy time (IT, 5.0 ± 0.48 min vs. PFNA-II, 2.8 ± 0.33 min), hospital stay (IT, 9.65 ± 0.95 days vs. PFNA-II, 8.58 ± 0.93 days), cut-out (IT, *n* = 0 vs. PFNA-II, *n* = 6), and tip-apex distance (IT, 26.7 ± 0.91 mm vs. PFNA-II, 23.2 ± 1.22 mm). No significant differences existed for the other observation indexes (*p* > 0.05).

**Conclusions:**

The IT nail may have more advantage for patients with unstable intertrochanteric fractures of the femur. However, for those complicated with lateral greater trochanter fractures, lateral cortex fractures of the proximal femurs, or unfit for surgery, the PFNA-II nail could be a good option. In addition, a large-sample, multicenter observational study is required for evaluation of its long-term efficacy, and optimal management strategies for specific unstable fracture patterns, different sorts of bone quality, and different levels of patient demand.

## Background

With the rapid increase in the elderly population, the morbidity of intertrochanteric femoral fractures (IFFs) is also displaying a rising trend [1–3]. And IFFs account for approximately half the hip fractures in elderly patients [4]. In order to return to pre-injury function and activity levels, surgical treatments have gradually become preferred. Currently, IFFs are usually treated with intramedullary fixations or extramedullary fixations [5]. Extramedullary fixations with advantages of less trauma, less bleeding, low levels of anesthesia, faster healing after surgery, and avoiding secondary operations had ever been considered the best choice in the treatment of IFFs. However, for unstable IFFs, the failure rate is higher, therefore IFFs are treated with intramedullary fixation devices (proximal femoral nail anti-rotation-Asia (PFNA-II), InterTan (IT)). Due to its lower failure and good biomechanical advantage that allows for immediate postoperative long-term, full-weight bearing of the hip, PFNA-II or IT is frequently used and has attained good clinical results in the treatment of unstable IFF in the elderly. PFNA-II, with a helical neck blade, is important in osteoporotic bone, and provides rotational and angular stability. IT, with the unique design of two cephalocervical screws in an integrated mechanism, allows linear intraoperative compression and rotational stability of the head or neck fragment. Two devices are being more widely used in clinics, particularly for the treatment of intertrochanteric fractures. However, it remains unclear which device provides better clinical and radiographic outcomes [6, 7].

The aim of this study was to compare the clinical outcomes of elderly patients undergoing surgery for treatment of unstable IFFs receiving either PFNA-II or IT.

## Methods

This study was approved by our responsible Investigational Ethical Review Board, and informed consents were obtained from patients or their authorized persons. Each patient was treated with PFNA-II or IT fixation devices (PFNA-II, proximal diameter, 16.5 mm; distal diameter, 9–10 mm; length, 240–280 mm; number of proximal/distal screw, 1/1; valgus curvature, 5°, Synthes, Solothurn, Switzerland. IT, diameter: lag screw, 11 mm; compression screw,7 mm; composite screw,15.5 mm; length, normal; number of proximal/distal screw, 2/1, Smith & Nephew, Memphis, Tennessee). The only intervention was ITs or PFNA-IIs of the unstable IFFs. The selection of the equipment size was according to the length of the femur, fracture types, the size of the medullary cavity, and bone mineral density (BMD). The number and combination mode of screws were also the same in each group. The inclusion criteria were as follows: fresh closed IFF (AO/OTA classifications 31-A2.1-3 and 31-A3.1-3), a low-energy trauma, age ranging 60–89 years. Exclusion criteria included multiple fractures, pathologic fractures, developmental dysplasia of the hip (DDH), clear contraindications, cases with severe medical disease, or American Society of Anesthesiologists (ASA) score of V. From January 2012 until June 2015, 168 patients with unstable IFFs treated with the PFNA-II or IT devices were enrolled in this study.

The trauma database in our medical center was investigated to identify demographics of all patients treated with ITs or PFNA-IIs. Patients eligible for inclusion were performed under the supervision of a trained orthopedic surgeon. No obviously abnormal results of cardiopulmonary function, blood gas analysis, chest X-ray, Hoher monitoring, echocardiography, and vascular ultrasound were recorded in each patient.

All patients were given a routine intravenous infusion of cefuroxime sodium 1.5 g 30 min before the skin incision was made and accepted general anesthesia. All of the operations were performed in the same institution by the experienced orthopedic surgeons. The technique was identical to that described by Mereddy et al. [8] for PFNA-II and Ruecker et al. [9] for IT. Good fracture reduction was obtained with the routine use of traction reduction, 10–15° adduction of the hip joint, and adjustment using a C-arm X-ray machine. Large-point reduction forceps or Kirschner-assisted reduction was used. In the proximal part of the greater trochanter, an approximate 4-cm small incision was made in each patient.

Postoperative treatment was essentially the same in both groups. Subcutaneous injection of Fraxiparine was administered once every day for 1 week to prevent deep vein thrombosis; within 2 days after the surgery, the conventional intravenous infusion of cefuroxime (3 g/day) was administered. At 24 h postoperatively, quadriceps contraction exercises were performed, and the patients were encouraged to exercise in bed. After 4–6 days, patients were allowed to out-of-bed activity. At 2–3 weeks postoperatively, the patients were encouraged to perform limb weight-bearing ambulation. After healing of the limb fracture was demonstrated by X-ray, full weight-bearing ambulation was demanded.

Operation time was measured as the interval from the start of the reposition to the wound closure. Fluoroscopy time was determined as the number of issues exposure, read on the fluoroscopy device at the end of the operation. The blood loss performed during or after the operation was recorded in terms of milliliters (mL). Bony union was defined as evidence of bridging callus or cortical continuity involving at least two cortices [10]. Tip-apex distance (TAD) was defined as the distance between the screw tip and apex of the femoral head in plain and lateral radiographs.

Statistical analyses were carried out using SPSS (SPSS statistic package, version 22.0.0) statistical software. The *T* test was used to determine whether there were any significant differences. The 2-tailed, unpaired *t* test was used to evaluate the differences between two groups, and the 2-tailed, paired *t* test was used to detect changes in preoperative to postoperative outcome scores. All continuous data are expressed as the mean ± standard deviation (SD). Independent sample t tests were used for the continuous variables. Categorical variables were analyzed with the chi-square test. The level of significance was set at *p* < 0.05 for all statistical analyses.

## Results

During the study, eight patients died (two in cerebrovascular accident, one in electric injury, four in lung cancers, and one in organophosphate poisoning), eight were too infirmed for follow-up, and seven were lost during follow-up; there were 147 patients remaining for the final evaluation. The mean follow-up time was 20 months (range 16–26 months). Perioperative-related data were shown in Table 1.

**Table 1:** Perioperative data of patients

Variable	PFNA-II group (*n* = 72)^a^	IT group (*n* = 75)^b^	*p*
Sex, no. M/F	32/40	35/40	0.787
Mean ± SD age, years	74.2 ± 9.1	75.2 ± 8.8	0.526
Side, no. left/right	38/34	43/32	0.579
AO/OTA fracture type, no.			
31A2.1	6	8	0.630
31A2.2	19	24	0.455
31A2.3	10	8	0.551
31A3.1	21	15	0.196
31A3.2	11	16	0.343
31A3.3	5	4	0.950
Mean operative time, minutes	52.3 ± 4.0	71.9 ± 6.8	0.000
Mean blood loss, mL	180.9 ± 10.8	190.6 ± 6.0	0.000
Mean fluoroscopy time, minutes	2.8 ± 0.33	5.0 ± 0.48	0.000
Mean hospital stay, days	8.58 ± 0.93	9.65 ± 0.95	0.000
TAD, mm	23.2 ± 1.22	26.7 ± 0.91	0.000

*Abbreviation*: *IT* interTAN nail, *PFNA*-*II* proximal femoral nail anti-rotation-Asia, *TAD* tip-apex distance

^a^Synthes, Solothurn, Switzerland

^b^Smith & Nephew, Memphis, Tennessee

With regard to complications (Table 2), significant differences existed regarding rotational loss of reduction (group PFNA-II vs. group IT, 9 vs. 0, respectively) (*p* = 0.005). Nine patients with lateral cortex fractures of the proximal femur or lateral greater trochanter fractures were recorded (group PFNA-II vs. group IT, 1 vs. 8, respectively) (*p* = 0.045). The occurrence of distal interlocking direction change was found in four cases, two in each group (*p* = 1.000), and the femoral head was penetrated by the lag screw in four patients (group PFNA-II vs. group IT, 3 vs. 3, respectively) (*p* = 1.000).

**Table 2:** Intraoperative and postoperative complications

Complications	PFNA-II group (*n* = 72)^a^	IT group (*n* = 75)^b^	*p*
Intraoperative complications	18	13	0.255
Improper placement of the device	1	4	0.388
Rotational loss of reduction	9	0	0.005
Penetrating the femoral head	3	3	1.000
Lateral greater trochanter fractures	1	8	0.045
Lateral cortex fractures of the proximal femur	1	8	0.045
Distal interlocking direction change	2	2	1.000
Local complications	20	10	0.030
Necrosis of the femoral head	2	3	1.000
Varus collapse of the head/neck	8	1	0.033
Femoral shaft fractures	8	1	0.033
Cut-out	6	0	0.033
Severe lateral migration of the hip screw	1	1	1.000
Implant failure	1	3	0.641
Pain of hip and thigh	17	7	0.019
Femoral neck shortening, mm	7.4 ± 2.4	4.4 ± 1.1	0.000
Mean healing time of bone	15.7 ± 2.4	14.7 ± 2.1	0.010
Mean ± SD HHS	83.8 ± 7.8	82.6 ± 7.1	0.302

*Abbreviation*: *IT* interTAN nail, *PFNA*-*II* proximal femoral nail anti-rotation-Asia, *HHS* Harris hip score

^a^Synthes, Solothurn, Switzerland

^b^Smith & Nephew, Memphis, Tennessee

Significant differences were also found regarding local complications: 20 (27.8 %) patients in group PFNA-II and 10 (13.3 %) in group IT (*p* = 0.030). Varus collapse of the head/neck and femoral shaft fractures at the tip of the nail were found in 8 vs. 1 cases in group PFNA-II vs. group IT, respectively (*p* = 0.033). Significant difference also existed with regard to hip and thigh pain (*p* = 0.019). Femoral neck shortening, which showed a significant difference (*p* = 0.000), was at an average of 7.4 mm (range 3–11 mm) in group PFNA-II and 4.4 mm (range 3–6 mm) in group IT. The mean bone healing time was 15.7 weeks (range 12–20 weeks) in group PFNA-II and 14.7 weeks (range 11–18 weeks) in group IT. The postoperative Harris hip scores (HHS) assessed at the 1-year follow-up was similar between the two groups; there was no significant difference between the final scores at both time points (*p* = 0.302). Also, no significant differences were found between the two groups with regard to the remaining symptoms evaluated.

## Discussion

The management of IFF has recently had much attention in the literatures [11, 12]. In the elderly population with unstable IFFs (intertrochanteric fractures involving the posteromedial walls or lesser trochanters), stable fixation is essential to allow early mobilization and to minimize the risk of morbidity and mortality. In order to increase the stability of the fracture fixation, some implants have been developed [13]. In the current study, intraoperative and postoperative complications with the PFNA-IIs and ITs were observed. Nevertheless, no significant difference existed between the two groups in the reduction results, implant positions, total complications, improper placements of the devices, penetrating femoral heads, distal interlocking direction change, necrosis of the femoral heads, severe lateral migration of the hip screws, implant failures, nonunion, malunion, delayed union, general complications, or postoperative HHS between the two groups. Furthermore, secondary surgical intervention was not required in any patient. Our findings are comparable with the results published in other studies [14–17]. However, the management of unstable IFF remains controversial, particularly in using PFNA-II or IT. The IT has become a standard treatment device. Its characteristics include two integrated screws with a hybrid worm-gear mechanism, a trapezoidal proximal end, an oval footprint, a “clothes-pin” distal tip, a unique geometry and mechanism of action, and initial linear compression, which prevent uncontrolled shortening during healing and varus collapse, thus improving rotational instability. The PFNA-II has a quite good anatomic fit, and its helical blade enhances bone purchase in the femoral neck–head. Additionally, the blade prevents rotation or compaction of the proximal fragment by locking with the nail rotationally. All these factors of the PFNA-II allow the patients to bear partial weight sooner after the surgery. Its disadvantages include cut-out and lateral migration of proximal screws or helical blades. Above all, main differences of both devices may be the abilities of compression and anti-rotation of the femoral neck–head in terms of performance, and single screw or double screws of the proximal femur in appearance. These differences may play a decisive role on surgical results.

The trapezoidal proximal end of the IT is difficult to insert into the narrow marrow cavity of Chinese patients, and the manipulation of repeatedly expanding the medullary cavity and repeated reduction may prolong the operative time, which in turn increases intraoperative blood loss and fluoroscopy time, especially in more unstable fracture types [18]. In the current study, the mean operative time, the mean blood loss, and the mean fluoroscopy time of the IT are larger than the PFNA-II. Based on these and complexity of inserting IT device, the PFNA-II may be more suitable for the frail patients.

The IT shows less inferior head displacement, higher loads to failure, and longer survival under physiologic loads compared to the PFNA-II. It might be explained by the fact that the two anti-rotational screws of the IT are positioned closer to the inferior femoral neck [19]; however, according to our research, it is more prone to lead to fractures of the lateral greater trochanter and lateral cortex of the proximal femur, although IT may decrease medialization by acting as a metal buttress [20]. This is rarely reported in the previous literatures [21, 22]. Some researchers believed that the main reason was inadequately expanding the medullary cavity, unsatisfactorily implant position, thin lateral wall, the loss of popularity for this device, or the poor supporting function of the lateral wall [9, 23]. Nevertheless, Huang et al. [22] reported that the IT nail was 30 % stronger than the PFNA-II in the proximal region. They believed that it was likely related to the proximal square cross-section, and the strengthening rotational stability in the marrow cavity that allowed it to compete with lateral stress and improve the supporting function of the lateral wall.

The PFNA-II, with the stress concentration caused by a poor contact with the bone from the lesser trochanter to the isthmus or the changeless diameter from the bending point to the distal tip, may easier result in the femoral shaft fractures. In contrast, the IT, with an ideal shape design of varying with the diameter of the proximal femoral medullary cavity, avoids stress overconcentration around the nail tip. Furthermore, the clothes-pin distal tip contributes to decreasing the overall cross-sectional stiffness of the implant distally, which may lead to better performance than that of PFNA-II.

Unstable IFF treated with an intramedullary device is commonly associated with mild pain. Lag screw cut-out or lateral protrusion may cause the long-term pain of the affected limb. And cut-out appeared to result from the poor positioning of the screw rather than being implant-related [24]. As reported by Takigami et al.[14], an inadequate insertion depth of the spiral blade and undelayed full weight bearing may result in cut-out, the incidence was 2 % in their study. Furthermore, when using only a single lag screw, the screw may serve as a type of rotational instability within the bone, and a secondary cut-out of the screw may occur with a loosening of the bone-screw interface caused by the flexion-extension of the limb. Cut-out rates ranged from 3 to 10 % [25]. Biomechanics-related and clinical studies have indicated that the ITs and PFNA-IIs have a good anti-rotation performance [23, 26]. In our studies, five (6.9 %) cases of cut-out in group PFNA-II and zero case in group IT were observed. Brunner et al. [27] reported three cases (2.3 %) postoperative cut-out of the blade through the femoral cortex occurred. The key of less cut-out was to make sure fracture reduction, the proper position of the screw, and the correct TAD [18]. In addition, the design of the implant may also be one of the effect factors resulting in cut-out [28, 29]. It is an undeniable fact that the one-screw anti-rotation of the PFNA-II has a certain limitation: once this limitation is exceeded, rotation of the head and neck may occur. Furthermore, the location of the spiral blade may not be in the lower one-third of the femoral neck. The self-drilling and smaller diameter screw would be more prone to migration through the femoral head, which could also increase the incidence of cut-out [30].

To a certain extent, TAD determines the success or failure of the operation, regardless of whether a 2- or 1-screw system is used, as stated by Nuchtern et al. [31]. In the study, despite significant difference regarding the TAD in both groups (23.2 ± 1.22 vs. 26.7 ± 0.91 in the PFNA-II and IT groups, respectively), no correlation existed between the TAD and functional outcomes. Indeed, TAD did not exceed in an acceptable range. It could be attributed to the fact that the extra time spent in inserting the proximal screws or the spiral blade in an ideal position on both AP and lateral view. For osteoporotic fracture in the elderly, although the screw with its tip up to the subchondral bone will achieve the best fixation effect, the shorter TAD may lead to cut-out of the internal fixation. Thus, the individualized principle is valuable. As for the group IT with higher TAD, we attribute it to the fact that in comminuted fractures, there is no complete bone block of the distal or proximal end for support, the pressure will be partially lost, and the intramedullary proximal combined nails of the IT sometimes cannot fully twist together, which may be related to the bone deficiency caused by comminuted fractures and the screw deviating from the intended trajectory during insertion.

Uncontrolled collapse can be prevented by the anti-rotation of the head and neck of the femur. It is also reported that the collapse can lead to neck malunion or unacceptable shortening of the head and neck segments [10, 25, 32]. Excessive shortening of the neck (>5 mm) may result in weakened strength of the gluteus medius and limit the movement of the hip joint [33]. To avoid shortening, the key point is to have the fracture reduced during the entire process from guidewire insertion through reaming, nail insertion, and locking [20]. The IT device, with a hybrid worm-gear mechanism converting rotational forces into linear compression, can overcome the shortening, which may be one of the main reasons why healing time is significantly shorter in group IT. However, no significant differences existed between the two groups regarding necrosis of the femoral head, lateral migration of the hip screw, implant failure, and final functional outcomes.

There were some limitations in this study. First, the follow-up was relatively short term. Second, this study was not a randomized trial. Third, the sample size of total patients was relatively small, which might have a certain influence on the results. Fourth, there were some subjective factors in the timing of postoperative weight bearing and the choice of intramedullary nail in the two groups.

## Conclusions

In summary, the results of our study show that the incidence of femoral shaft fractures, rotational loss of reduction, varus collapse of the head/neck, pain of hip and thigh, cut-out, and femoral neck shortening were decreased in group IT comparing with group PFNA-II. The IT may have more advantage for patients with unstable intertrochanteric fractures due to its low complication rates. However, for those complicated with lateral greater trochanter fractures, lateral cortex fractures of the proximal femur, or unfit for the surgery, the PFNA-II could be a good option. In addition, a large-sample, multicenter observational study is required for evaluation of its long-term efficacy, and optimal management strategies for specific unstable fracture patterns, different sorts of bone quality, and different levels of patient demand.

## Abbreviations

ASA: anesthesiologists
DDH: developmental dysplasia of the hip
DHS: dynamic hip screw
HHS: Harris hip score
IFF: intertrochanteric fractures of femur
IT: InterTan nail
PEFFs: proximal extra-capsular femoral fractures
PFNA-II: proximal femoral nail anti-rotation-Asia
SD: standard deviation
SPSS: Statistical Product and Service Solutions
TAD: tip-apex distance
